# Assessment of post-pandemic NAAT-based diagnostic capacity among laboratories with COVID-19 testing resource investments in Indonesia

**DOI:** 10.1371/journal.pone.0343628

**Published:** 2026-04-02

**Authors:** Bony Wiem Lestari, Almira Alifia, Putu Wahyuni Wulandari Karnawati, Nurlaila Syahri Ramadhani, Selvia Kusdwiyanti, Nurholis Majid, Ryan Bayusantika Ristandi, Rifky Waluyajati Rachman, Philip C. Hill

**Affiliations:** 1 Research Centre for Care and Control of Infectious Diseases, Padjadjaran University, Bandung, Indonesia; 2 Department of Public Health, Faculty of Medicine, Padjadjaran University, Bandung, Indonesia; 3 West Java Provincial Health Office, Bandung, Indonesia; 4 Association of District Health Laboratories in West Java, Bandung, Indonesia; 5 West Java Public Health Laboratory, Bandung, Indonesia; 6 Center for International Health, University of Otago, Dunedin, New Zealand; Waseda University: Waseda Daigaku, JAPAN

## Abstract

**Background:**

Throughout COVID-19 pandemic, many countries expanded their diagnostic infrastructure, including Indonesia. West Java, one of the most COVID-19-affected provinces in Indonesia, received many PCR machines during the pandemic but the molecular diagnostic landscape left post-pandemic needs to be assessed to build an improved pandemic preparedness in the area and enhance control of other diseases.

**Aims:**

To evaluate the post-pandemic diagnostic capacity of district health laboratories (DHLs) in West Java, to determine factors associated with laboratories’ performance capacities and to portray its DHLs’ diagnostic profiles to explore the possibility of implementing an additional NAAT-based tuberculosis test in the province.

**Methods:**

This was a cross-sectional laboratory survey, assessing 26 DHLs in West Java, Indonesia. Data were collected through direct visits, interviews based on questionnaire based on WHO Laboratory Assessment Tool, and focus group discussion. The questionnaire was then quantitatively scored and used to categorize capacity levels as strong (>=85%), good (70–84%), weak (50–69%), or very weak (<50%). Descriptive statistics were also used to highlight key gaps.

**Results:**

Out of 26 DHLs, only 34.6% had strong NAAT-based diagnostic capacity, 34.6% had good capacity, while 30.8% were categorized as weak or lower. 80.8% of the DHLs scored weak or very weak in ‘Laboratory Testing Performance’ module, and only 11.5% of DHLs conducted PCR machine calibration in the past year. Only 46.2% maintained a functional BSL-2 laboratory, and the median of PCR-trained staff was 2 (IQR 1–4), with 15.4% DHLs no longer retaining any biomolecular staff.

**Conclusion:**

The sustainability of COVID-19 diagnostic investments post-pandemic is relatively poor across many districts in West Java. Commitment to sustain facilities and equipment, as well as to retain an adequate workforce, is warranted to strengthen pandemic preparedness in the area and to repurpose COVID-19 facilities for tuberculosis control purposes.

## Introduction

The COVID-19 pandemic underscored the critical need for fundamental preparedness in addressing global health emergencies. Preparedness involves establishing robust public health systems capable of rapid surveillance, early detection, and effective response [1]. Significant investments were made towards pandemic management, including in molecular diagnostic infrastructure. Indonesia mandated 685 number of laboratories for COVID-19 testing in the first year of pandemic [2]. Nucleic Acid Amplification Test (NAAT)-based diagnostic machines were distributed to health laboratories, including those in West Java province, one of the most impacted provinces with 1.1 million of COVID-19 cases in 2023 [3].

As the demand of COVID-19 testing has declined in Indonesia, sustainability of the NAAT machines dedicated for COVID-19, like its availability or operationability post-COVID, is largely unknown. Sustaining these diagnostic machines can be crucial for pandemic preparedness, but NAAT-based diagnostic machines can also be reallocated for use against other diseases to fill gaps in diagnostic capacity. An obvious candidate is tuberculosis (TB) – in 2024, Indonesia was reported to have the second highest burden of TB in the world, with an estimated incidence of 382 per 100,000 population and caused 125,625 deaths [4]. Furthermore, despite programmatic improvements by the Indonesian government for TB control, gaps in TB diagnostic capacity remain [4]. In 2024, the global size gap between estimated incident cases of TB each year and the officially reported TB cases was around 2.7 million [4]. Efforts are needed to improve the early case detection, leading to early treatment. The government has implemented nationwide provision of GeneXpert rapid molecular tests as the primary diagnostic test for TB, but capacity to meet the need has been strained at times [3,5]. Southeast Asia still has a relatively small number of coverage of GeneXpert testing. Only 39% patients with new or relapse TB were tested with a WHO-recommended rapid diagnostic (WRD) in 2023 [6].

Several calls have been published to utilize previous COVID-19 diagnostic tools for other diseases and to build stronger pandemic preparedness, but capacity mapping exercises in any setting are rare [7,8]. Hence, this research aimed to evaluate NAAT-based diagnostic capacity of health laboratories in West Java post-pandemic, to identify factors associated with laboratories’ performance capacities and to map the laboratories’ diagnostic profiles to explore the opportunity to enhance NAAT-based TB test services.

## Materials and methods

This study employed a predominantly quantitative cross-sectional laboratory survey design to assess the laboratory capacity across district health laboratories (DHLs) in West Java. Participants recruitment and data collection were conducted between May 30^th^, 2024 and December 31^st^, 2024 using structured questionnaires, interviews, and direct observations that were based on WHO Laboratory Assessment Tools (LAT) [9]. The qualitative part of this study was carried out using a sequential explanatory design, using focus group discussions (FGD) to explore enablers and barriers to test capacity transition.

### Study setting

West Java Province is the most populated province in Indonesia, with more than 48 million inhabitants in 2020 [10]. West Java has 27 districts, 26 of whom have a DHL, spanning across area of 35,378 km^2^ [11], and was also one of the most impacted provinces by the COVID-19 pandemic despite underreporting issues [12]. Data and contact of all 26 DHLs were gathered through the West Java Provincial Health Office (PHO). The majority of DHLs received an NAAT-based diagnostic tool in the form of PCR machine during the COVID-19 pandemic.

### Data collection

We designed and administered a short questionnaire to all 26 DHLs about availability of PCR machines in their laboratories, the brand and function of their machines, and availability of PCR service at the present time. We did independent visitation to all DHLs to do direct observation and a structured interview using a questionnaire developed based on WHO LAT, which is a standardized WHO laboratory assessment tool [9] containing a number of modules and indicators that can be adapted by public health practitioners to evaluate specific laboratory capacity [9]. For this assessment, we selected 9 out of 11 WHO LAT modules (Organization and Management, Human Resources, Facilities, Equipment, Consumables, Specimen Handling, Biorisk Management, Laboratory Testing Performance, and Data and Information Management). The modules were then adapted to the local context, through modified wording to improve interpretation, simplified questions to avoid poor engagement during data collection, and translation into Indonesian. We also interviewed PCR-trained staffs, asking them for the level of agreement to specific statements regarding PCR diagnostic testing. There were four statements and they can choose their level of agreement for each statement from strongly disagree, disagree, neutral, agree, or strongly agree. The questionnaires used in this study is available in S1 File.

We organized an online FGD to discuss the underlying challenges faced by DHLs on several specific parts of the WHO LAT modules that are considered low-performing. The participants of the FGD were DHLs’ heads and laboratory staffs to give information on that topic.

### Data analysis

A flowchart diagram was made to describe current NAAT-based diagnostic landscape in West Java, based on the answers to the short questionnaire. The questionnaires were adapted from WHO, with adaptations made in accordance to local context. Validation was not needed as the questionnaires have descriptive purposes. We filtered out questionnaires according to WHO LAT guidance to do scoring analysis. Descriptive statistics were used to analyze the data, with results presented in terms of frequencies, percentage, and median, along with the scoring of the LAT questionnaire. The scoring was done by treating the answer of each closed question as a binary number (0/1). In the Facilities module, score of 0.5 of “Availability of BSL-2 facility” questions reflected that the said facility is still in development. Meanwhile, for ordinal categorical-type options, as such in the Facilities and Consumables modules, each option was ordinally valued as 0, 0.33, 0.67, and finally 1. All questions’ score in each module were calculated and converted into a percentage to present the score for each module for each DHL. An overall score for each DHL was determined by calculating the average percentage score of all modules. We categorized module scores based on categorization used by Liu et al: strong (85% or more), good (70–84%), weak (50–69%), and very weak (less than 50%) [13].

We performed a chi-square test to identify the key indicators associated with laboratory performance, and p-value of <0.05 was considered statistically significant. Descriptive statistics were also used to summarize biomolecular staff perceptions about PCR diagnostic methods. There were four statements and the staff may choose their level of agreement for each statement from strongly disagree, disagree, neutral, agree, or strongly agree. The questionnaires used in this study are available in S1 File. A thematic analysis was done to summarize FGD results. The FGD was recorded, which was then transcribed verbatim and coded using ATLAS.Ti (Lumivero, LLC) software.

### Ethics approval and consent to participate

Ethical approval for this study was obtained from the Research Ethics Committee Universitas Padjadjaran Bandung (578/UN6.KEP/EC/2024). Written informed consent was obtained and signed by all participants prior to the commencement of data collection.

## Results

### Current NAAT-based diagnostic landscape

**Fig 1:**
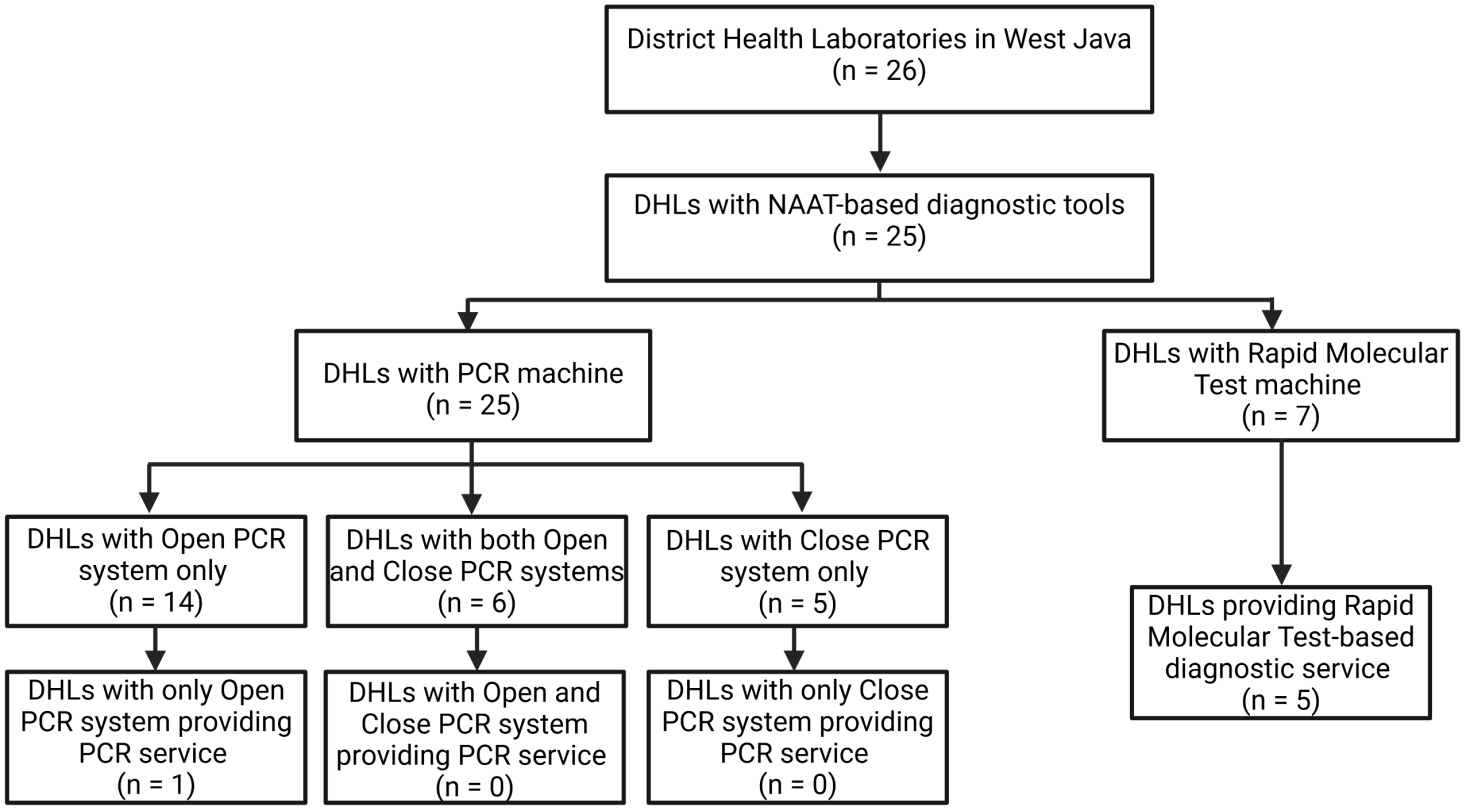
Profile of NAAT-based diagnostic tools and service of the assessed DHLs.

Regarding machine and service availability, there was one DHL that did not own any NAAT-based diagnostic tools. A detailed flowchart of this matter is shown in Fig 1. Of the remaining 25 DHLs, only 7 (28%) owned a Rapid Molecular Test (RMT) machine, either a GeneXpert® from Cepheid or Truenat® machine. An even smaller proportion of them (20% (5/25 DHLs) currently provide an RMT-based diagnostic service. In terms of PCR machines, all of the 25 DHLs owned at least one, although each might have a different system and brand. 84% of the PCR-owning DHLs had an open PCR system, and 6 DHLs had both open and closed PCR systems. Among all DHLs, only one DHL retained a PCR service until the period of this study.

### Health laboratory capacity

**Fig 2:**
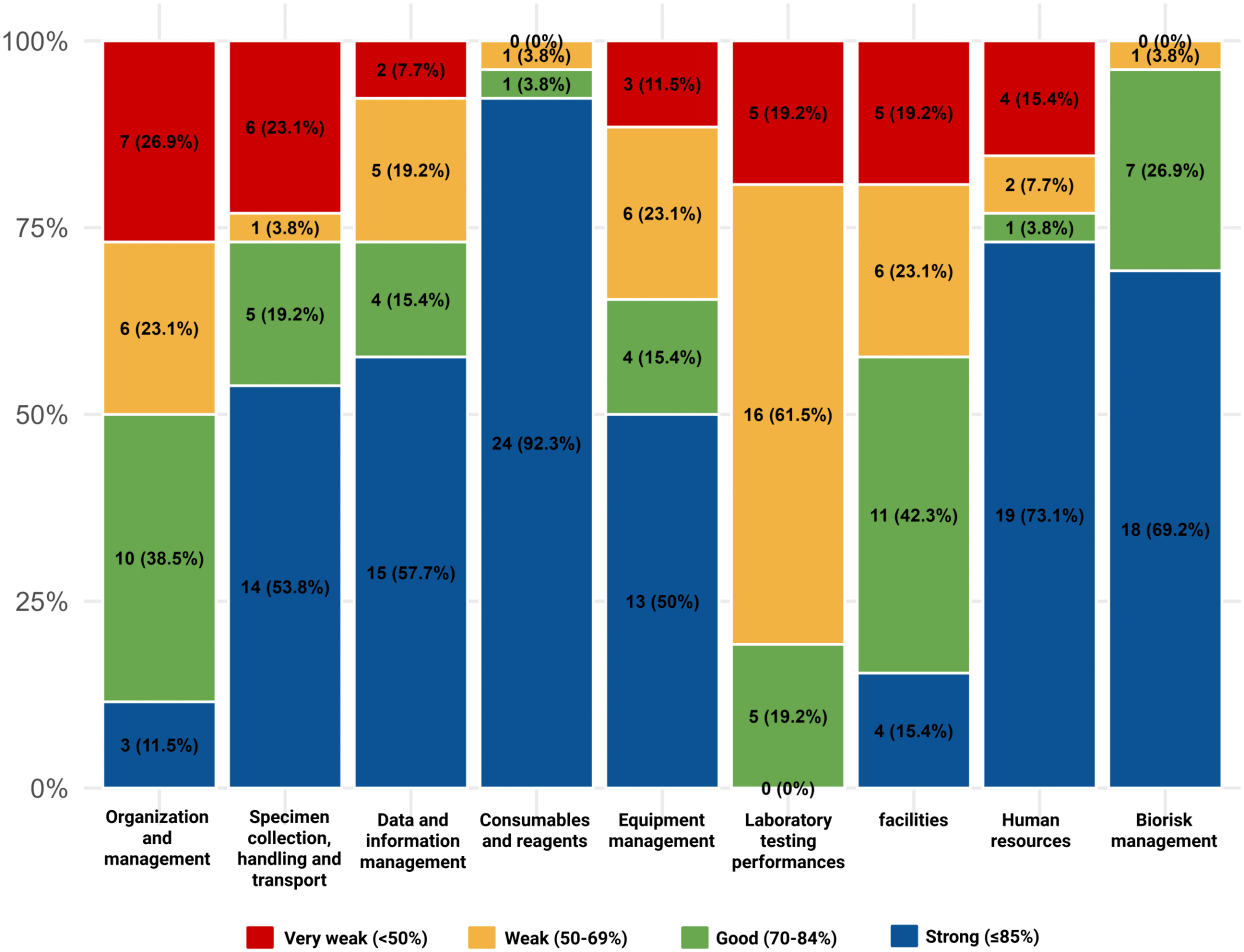
Stacked bar chart of DHLs’ performance in each assessment module.

Of 26 DHLs, only nine (34.6%) had strong (>= 85%) NAAT-based diagnostic capacity. Nine (34.6%) were categorized as good, seven (26.9%) were weak, and one (3.9%) was very weak. The average score of 9 categories reflecting the laboratory capacities, from across all DHLs, was 75.4%. A more detailed breakdown of the scoring proportion in each module is shown in Fig 2, and the dataset gathered for this analysis is available in S2 File.

The module with the highest number of ‘very weak’ and ‘weak’ scores was Laboratory Testing Performance. Among those with PCR machines, many no longer had available usable reagents or calibrated their PCR machine, which explains the low scores.

The best performing module in all laboratories was Consumables and Reagents, which 24 laboratories (92.3%) scored as ‘strong’. The majority of the laboratories had a good inventory system of consumables, appropriate storage for reagents, and strict use of non-expired reagents. More highlighted aspects from each module are shown in Table 1. There are several aspects that were majorly unavailable in many laboratories, such as ISO accreditation (ISO 17025:2017 about Laboratory Testing and Calibration Competency), which 73.1% of DHLs did not have. Similarly, 73.1% of the assessed DHLs also did not possess a laboratory information system, and 69.2% did not have a −80 °C freezer. Only 65.4% of the DHLs owned a BSL-2 laboratory, although there were six (23.1%) who had one under construction. Only 46.2% (n = 12) of the DHLs had a functional BSL-2 laboratory at the time of assessment. Additionally, a limited pool of staff trained in PCR (median = 2) was observed and the complete descriptive statistics of all aspects from the assessment module is available in S3 File.

**Table 1:** Descriptive statistics of several highlighted aspects from each assessment module (N = 26).

Module		Aspect	Yes (n, %)
**A.**	**Laboratory Management**	1. Sufficient budget assigned for consumable and reagent purchase	17 (65.4)
		2. Sufficient budget assigned for equipment purchase and maintenance	14 (53.8)
		3. Accreditation of ISO	7 (26.9)
**B.**	**Human Resources**	1. Staff qualification	**Median (IQR)**
		- Laboratory technician	7 (4–9)
		- Trained staff to do PCR	2 (1–4)
		2. Training	**Yes (n, %)**
		- Molecular testing	20 (76.9)
		- Real-time PCR	18 (69.2)
**C.**	**Laboratory Facilities**	1. Availability of BSL-2 laboratory	17 (65.4)
		2. Functional BSL-2 laboratory	12 (46.2)
		3. Availability of tri-room separation (preparation, extraction, and amplification)	18 (69.2)
		- Refrigerator/chiller	21 (80.8)
		- Freezer -20^o^C	14 (53.8)
		- Freezer -80^o^C	8 (30.8)
**D.**	**Equipment Management** **(N = 25)**	1. Availability of equipment condition’s inventory	19 (73.1)
		2. Availability of equipment calibration’s protocol and scheduling	18 (69.2)
**E.**	**Specimen collection, handling, and transport**	1. Availability of specimen record system	20 (76.9)
		2. Availability of procedure for specimen storage post-analysis	18 (69.2)
**F.**	**Consumables and Reagents Management**	1. Appropriate storage of consumables and reagents with temperature and humidity monitoring	23 (88.5)
		2. Availability of a system for accurately forecasting needs for consumables and reagent	23 (88.5)
**G.**	**Laboratory testing performance**	1. PCR	
		- Competent staff to perform the test	20 (76.9)
		- Machine calibration in the past year	3 (11.5)
**H.**	**Biorisk Management**	1. Biosafety training	17 (65.4)
		2. Availability of management procedure for infectious and non-infectious waste	26 (100.0)
**I.**	**Data and Information** **System**	1. Access protection for patient data	24 (92.3)
		2. Patient result data backup	18 (69.2)
		3. Availability of laboratory information system (LIS)	7 (26.9)

Over three-quarters (76.9%, n = 20) of the DHLs had provided molecular test training to their relevant staff. Equipment condition’s inventory and protocol were available in more than half of the DHLs, and 76.9% (n = 20) of the laboratories had a specimen record system in place. All laboratories also had infectious waste management procedures available. Despite the unavailability of LIS across laboratories, 92.3% (n = 24) had protection of access for patient data and 69.2% (n = 18) had a backup system for patients’ test results. The association of key indicators per laboratory modules with laboratory performance capacities is presented in Table 2.

**Table 2:** Comparison of key indicators by laboratory performance (N = 26).

Module	Aspect	Very weak n = 1 (3.8%)	Weak n = 7 (26.9%)	Good n = 9 (34.6%)	Strong n = 9 (34.6%)	p-value
Laboratory management	Sufficient operational budget	0 (0.0)	4 (57.1)	4 (44.4)	6 (66.7)	0.55
	Accreditation status	0 (0.0)	0 (0.0)	2 (22.2)	5 (55.6)	0.08
Human resources	Molecular testing training	0 (0.0)	2 (28.6)	9 (100.0)	9 (100.0)	<0.01
	Real-time PCR training	0 (0.0)	1 (14.3)	8 (88.8)	9 (100.0)	<0.01
Laboratory facilities	Functional BSL-2	0 (0.0)	3 (42.9)	3 (33.3)	6 (66.7)	0.39
	Refrigerator/chiller	1 (100.0)	5 (71.4)	7 (77.8)	8 (88.8)	0.79
	Freezer -20^o^C	0 (0.0)	4 (57.1)	4 (44.4)	6 (66.7)	0.55
	Freezer -80^o^C	0 (0.0)	1 (14.3)	2 (22.2)	5 (55.6)	0.24
Equipment management	Equipment inventory	0 (0.0)	5 (71.4)	6 (66.7)	8 (88.8)	0.26
	Equipment calibration’s protocol and scheduling	0 (0.0)	5 (71.4)	5 (55.6)	8 (88.8)	0.20
Specimen collection, handling, and transport	Specimen record system	0 (0.0)	2 (28.6)	9 (100.0)	9 (100.0)	<0.01
	Procedure for specimen storage	0 (0.0)	2 (28.6)	8 (88.8)	8 (88.8)	0.01
Consumables and reagents management	Storage with temperature and humidity monitoring	1 (100.0)	6 (85.7)	8 (88.8)	8 (88.8)	0.98
	Availability of a system for accurately forecasting the needs	1 (100.0)	5 (71.4)	9 (100.0)	8 (88.8)	0.35
Laboratory testing performance	Availability of PCR-trained staff	0 (0.0)	4 (57.1)	9 (100.0)	9 (100.0)	<0.01
	PCR machine calibration in the past year	0 (0.0)	1 (14.3)	0 (0.0)	2 (22.2)	0.50
Biorisk management	Biosafety training	1 (100.0)	3 (42.9)	5 (55.6)	8 (88.8)	0.20
	Availability of management procedure for infectious and non-infectious waste	1 (100.0)	7 (100.0)	9 (100.0)	9 (100.0)	NA
Data and information system	Access protection for patient data	1 (100.0)	6 (85.7)	9 (100.0)	8 (88.8)	0.70
	Patient result data backup	0 (0.0)	2 (28.6)	7 (77.8)	9 (100.0)	<0.01
	Availability of laboratory information system (LIS)	1 (100.0)	1 (14.3)	2 (22.2)	3 (33.3)	0.31

Of the nine domains included in the quantitative survey, six were then explored further through the FGD, as described below.

### Perceptions on PCR

At the time of the study, 84.6% of DHLs (n = 22) had at least one PCR-trained staff, while other DHLs had no PCR-trained personnel. We interviewed one PCR-trained staff from each of the 22 DHLs. Of these, 15 (68.1%) disagreed or strongly disagreed that the PCR diagnostic method is difficult to do. Similarly, 12 (54.6%) disagreed or strongly disagreed that PCR is troublesome. The majority (90.9%, n = 20) of interviewees agreed or strongly agreed that the method needs trained personnel to do so. However, despite the majority of the representing staff agreed or strongly agreed that their laboratories are ready to use PCR if it is available (95.5%, n = 21), one interviewee strongly disagreed that their laboratory is ready to use the method (4.5%).

### Barriers of laboratories’ performance

#### Laboratory management.

Our study showed only 7 out of 26 DHLs (26.9%) have accreditation for ISO. Through FGD, it was found that DHLs had difficulties in meeting accreditations’ criteria. The challenges included having to be updated with more recent standards for machine calibration, clashes with building renovations, and insufficient human resources. Budget constraints for accreditation preparation was also mentioned, although these were reduced once the DHL had achieved accreditation. Lack of information regarding accreditation itself was mentioned as one of the barriers of DHLs to participate in accreditation schemes.

“*Our DHL have followed ISO accreditation since 2013, and since I have been working here from 2015 (I have) participated twice on the survey.. And did surveillance several times. And indeed in pursuing accreditation there must be a lot of challenges from unmet standards.. There were several uncalibrated machines as the vendor could not find (the calibrator).. To unrenewed methods because there was an updated standard of method but we could not get the new standard.. and then about the room condition that was indeed needing a repairment.. and there is no accreditation that can be achieved easily, everyone must go through that and it is (necessary) to make our laboratory better, so there’s that.*” – Head of DHL B

#### Human resources.

To retain a sufficient number of PCR-trained staff, DHLs expressed various challenges post-pandemic. The median of trained staff to perform PCR was 2 (IQR 1–4), meanwhile there were four DHLs with no PCR-trained staff. There were several new regulations regarding limits of middle-level staff in each laboratory, and some government-employed staff were ordered to move to other institutions. Reduced funding post-pandemic meant that only a few staffs can be retained. Different lab status also influenced how each DHL was funded, which could affect staff retainment and recruitment as well.

“*For us, previously human resources (were paid) from the fund.. the COVID special fund, yes.. Previously we had administrative (staff), (so) in total there were five of us. And then..... because the pandemic has ended, so it (the COVID special fund) no longer exists, so in PCR laboratory the remaining staffs are just the two of us.*” – Lab Technician from DHL I

#### Laboratory facilities.

17 out of 26 DHLs (65.4%) have certified as a BSL-2 laboratory, but only 46.2% (12/26) have a functional BSL-2 laboratory. Operationality of BSL-2 labs post-pandemic were halted for multiple reasons. These included reagent shortage, inadequate electrical power, and not enough money for maintenance.

“*The situation in our DHL is that our BSL have not been operational for around two years. It is being operated sometimes, but recently we rehabilitated our lab rooms so for now the BSL-2 is not being used because there is not enough electrical power.. But hopefully the plan is next year we can cover that. With the planned electrical power we hope it can funtion (the BSL-2), because indeed the challenge is the enormous need of the electricity for this BSL-2 facility, and the maintenance (fee) is quite (expensive) too for BSL-2.*” – Head of DHL B

#### Laboratory testing performance.

Continuity of PCR machine calibration was hindered for several reasons across DHLs, including lack of budget to prioritize it among other necessities, rare use of machines post-pandemic, unavailability of calibration tools, and administrative challenges posed by calibration system registration and the machine’s procurement source. It is supported by the fact that 69.2% (18/26) DHLs have the availability of equipment calibration’s protocol and scheduling.

“*The challenge regarding PCR is that-- ours is one of the machines that is not calibrated yet. While for other tools like pipettes, coolers, freezers, photometry machines, etc all have been calibrated. But for PCR is not yet (calibrated), due to (lack of) budget because (PCR) calibration is quite expensive, around 20 million (rupiahs). While from our budget, that 20 million can be used for (maintaining) other smaller tools in the lab, (it could cover) up to 10 or 15 items. So we prioritize those.*” – Head of DHL’s A

#### Biorisk management.

Of 26 DHLs, 17 (65.4%) have already undergone biosafety training. Provision of biosafety training was seen as difficult in several DHLs because they had limited human resources to back up the workload, and/or limited funds for overall capacity building for laboratory staff.

“*Last year I joined a biosafety training in Jakarta if I am not mistaken.. For the next year there has not been any (similar program) yet.. So for us to join (such kind of program) independently, we’re quite challenged on..the budget. But if there is a training from.. provided by the MoH for instance.. we would join it.*” – Lab Technician from DHL L

#### Data and information system.

Perceived high costs of developing an LIS were mentioned several times by different DHL representatives. It was mentioned that there was no budget to develop LIS, and that they prefer to wait for an initiative from MoH for such a system. Additionally, having an LIS was seen as eventually adding to staff workload, because they would have to keep both physical and digital records instead of dealing with digital records only. This burden was captured on the availability of LIS on only 26.9% (7/26) DHLs.

“*Hence in our place so far there is no (LIS).. … so far our capability is limited because when we consulted with vendor for developing such a system application, it could cost until tens of millions, yes, so we are unable to cover that and hence with a heavy heart we just wait for the one launched by the MoH.*” – Head of Administration from DHL F

## Discussion

Across nine domains of evaluation, we found that after the COVID-19 pandemic, many DHLs in West Java that were equipped with resources for COVID-19 testing were no longer demonstrating adequate capacity to deliver NAAT-based testing. Many did not have a functional BSL-2 laboratory, and PCR machines were poorly maintained. Budget was mentioned to be one of the key barriers to retaining laboratory performance post-pandemic. The study reveals key gaps to sustain COVID-19 investments, which pose a threat to the province’s pandemic preparedness. Throughout COVID-19 pandemic era, NAAT equipments were shifted to enhance mass diagnostic effort. Ministry of Health (MoH) of Indonesia had already arranged 45 referral laboratories in 22 out of 34 provinces during the beginning wave of COVID-19 (April 2020) era in Indonesia. It then increased to 685 laboratories across 34 provinces by one year since the first case of COVID-19 was diagnosed. The laboratories were prepared with only PCR machines, only RMT machines, or both. At that moment, West Java was one of the provinces with the highest number of laboratories at the ready. It is acknowledged that unequal distribution of appropriately trained human resources was still a challenge [2]. An effort has been made to develop an open system of real time PCR (RT-PCR) assay machine which could be applied on the COVID-19 facilities, whose results reporting reliable sensitivity of drug resistance/multi-drug resistance (DR/MDR) RT-PCR on detecting Mycobacterium tuberculosis (MTB), rifampicin (RIF), and isoniazid (INH) resistance on a par with culture and drug sensitivity test (kappa value ≽0.8) [14].

Indonesia progressed well in upscaling one of the molecular WHO-recommended rapid diagnostic (mWRD) low complexity NAATs, GeneXpert, in 1.944 health facilities of 500 districts by January 2023. However, only 16.9% of GeneXpert health facilities are operational among 11,483 microscopic health facilities [15]. Only 66% of the reported cases were tested with a mWRD, and only 51% of reported cases were confirmed bacteriologically [16]. It is imperative to make proper arrangements for NAAT machines, including those previously used for COVID-19 testing, to increase TB case diagnosis.

The findings of this study resonate with the scoring by Global Health Security Index, which scored Indonesia’s Laboratory Systems and Quality at 75% [17], reflecting the national capacity to detect priority diseases and identify outbreak pathogens [18]. The findings of this study underscore the need to capitalize on the major government investment in DHL molecular diagnostic testing capacity during the pandemic by optimising the approach to sustaining it.

This study highlights that maintenance of facilities and equipment, in addition to the availability of LIS, which are needed to support NAAT-based testing, seems to be burdensome to retain post-pandemic because of limited funding from local governments. Proper preventive maintenance can improve reliability and reduce operational costs, as demonstrated by predictive maintenance systems used in some countries, saving significant resources [19]. Poor maintenance of equipment is linked to shortage of specifically trained technical staff [19], as confirmed by our study. Shortages of trained technical staff also limits a laboratory’s capacity to meet the diagnostic needs of an outbreak response [20]. Our findings align with the challenges reported in other resource-limited settings. A study in Ethiopia found that only 40% of laboratory staff were trained in advanced molecular testing, which negatively impacted diagnostic efficacy [21]. Another study in India revealed that PCR-trained personnel were only present in tertiary care centers, leaving peripheral laboratories ill-equipped for PCR-based diagnostics [22]. Furthermore, most of the laboratories were not equipped with the LIS, which might be related to inadequate safeguarding of patient data, in particular, patients’ confidentiality. LIS plays a role in facilitating communication between healthcare providers and affiliated laboratories, serving as a decision support system. LIS data also functions to improve patient safety as it provides data from pre-test, pre-analytic, analytic, post-analytic, and post-test phases. That being said, LIS works as a monitor for antibiotic resistance [23]. The unavailability of a data backup system may cause inefficiency due to the possibility of repeated testing, because every sample will be tested at the physician’s request.

With respect to the plan of repurposing COVID-19 PCR machines for TB diagnostics which was recently enforced by a national recommendation [5], it is clear that a number of challenges need to be overcome. Considering a generally positive view on PCR diagnostic methods by current biomolecular staff, the laboratory repurposing might be easier to implement, although refresher trainings and additional laboratory officers as well as establishment of LIS would be needed. Diagnostic tools repurposing was achieved by multiple countries early in the pandemic to support COVID-19 testing [24,25]. The reverse should be achievable.

This is the first study to assess the NAAT-based diagnostic testing capacity of DHLs in the aftermath of the COVID-19 pandemic in Indonesia. Furthermore, this study explored the underlying barriers and possible enablers that could better explain the situation. We also linked that assessment to the plan of repurposing COVID-19 diagnostic tools for TB. The study has also mapped DHLs with available open PCR system, which could ease the process of repurposing those for TB diagnostics. The strong collaboration with the Provincial Health Office (PHO) of West Java enabled us to gather reliable data and maintain close connections with their officials.

However, this study has several limitations. First, not all modules in WHO LAT were included in the questionnaire. However, the modules that were taken out (Documents and Public Health Functions) were considered not particularly relevant to the aims of this study. Secondly, the use of a specific tool to assess laboratory capacities (WHO LAT) serves as a guide to classify the results, which may hinder its applicability in a real setting. Therefore, for refocusing of NAAT machines previously used in the COVID-19 era for TB testing in our setting, we would certainly apply stronger and stricter criteria, such as functional BSL-2 facilities, tri-room separation, recent calibration, and documented standard operating procedures (SOPs), to ensure operational readiness. Third, due to the researchers’ collaboration with Provincial Health Office, interviewees may have seen the researchers as making assessments on behalf of their direct superiors. This risk of bias was mitigated by doing independent visits to the DHLs without government officials and observing supporting data from other aspects of the visits. Lastly, as our study setting was confined to West Java province, this limits the generalisability of the findings.

Based on the findings of this study, public health officials are encouraged to address the gaps in order to improve pandemic preparedness and TB control in the area. The necessity of adequate funding, equipment maintenance, and sufficient numbers of trained biomolecular staff are particularly emphasized. Further studies might assess more laboratories across the country, and identify options to improve the capacity of health laboratories where large gaps were identified.

## Conclusion

The COVID-19 pandemic led to multiple investments that can contribute to better pandemic preparedness and enhancing control of other diseases in West Java, Indonesia, but sustaining these investments is challenging. NAAT-based diagnostic equipment is in need of more routine maintenance, and more support is needed to sustain lab facilities and trained workforce. This study has identified opportunities for improving pandemic preparedness and repurposing COVID-19 tools for TB diagnosis, while also identifying gaps that need to be filled for successful implementation.

## Supporting information

S1 File Questionnaire. (PDF)

S2 File Scoring of Laboratory Assessment Tool Questionnaire. (PDF)

S3 File Descriptive statistics of all aspects from the assessment module. (PDF)
