# Supplementary material for: Assessment of post-pandemic NAAT-based diagnostic capacity among laboratories with COVID-19 testing resource investments in Indonesia
Source: PLoS One. 2026 Apr 2;21(4):e0343628. doi: 10.1371/journal.pone.0343628 (PMC13046156; doi:10.1371/journal.pone.0343628)
Supplement: S1 File — (PDF) [file pone.0343628.s001.pdf]

Supporting Information 1: Questionnaires

Laboratory Assessment Tool/Questionnaire

**Assessment of post-pandemic NAAT-based diagnostic capacity among laboratories with COVID-19 testing resource investments**	
Date of the assessment:	Name of the Assessor:

**1. Laboratory Identification**		
1.	Province	
2.	District/City	
3.	Name of the laboratory	
4.	Address	
5.	Telephone	
6.	E-mail	
7.	Type of laboratory	☐ Community health center<br>☐ Hospital-based<br>☐ District Health<br>☐ Private-based
8.	Level of laboratory	☐ Tier 1 (Peripheral)<br>☐ Tier 2 (District)<br>☐ Tier 3 (Intermediate)<br>☐ Tier 4 (Regional)<br>☐ Tier 5 (Central)
9.	Status of laboratory	☐ BLUD<br>☐ Non BLUD
10.	Is PCR COVID-19 testing available in place?	[ ] Yes<br>[ ] No
11.	PCR machine	
12.	Type of PCR machine	[ ] Open system<br>[ ] Closed system
13.	Status of PCR machine procurement	[ ] Local government budget<br>[ ] National government budget<br>[ ] Grant<br>If "Grant", please describe below:
14.	Describe participation in national programmes (if applicable)? (Check relevant box/es)	☐ Tuberculosis<br>☐ HIV<br>☐ Malaria

2. Assesment of TB testing capacity				
1.	Is the laboratory able to perform one or more of the following activities?	**Yes**	**No**	**Notes**
	a. Sputum collecting and handling			
	b. AFB smear test			
	c. Solid culture			
	d. Liquid culture			
	e. Phenotypic drug susceptibility testing (pDST)			
	f. Minimum Inhibitory Concentration (MIC)			
	g. Targeted Next Generation Sequencing			
	h. Whole Genome Sequencing			
2.	Is the GeneXpert machine available in place?	**Yes**	**No**	**Notes**<br>*If yes, continue to question 2.a-2.c*
	a. GeneXpert MTB/Rif test			
	b. GeneXpert MTB/Rif Ultra test			
	c. GeneXpert MTB/Rif XDR			
3.	How many sputum samples from suspected TB patients from community health centers, private clinics, and/or hospitals have been examined at this facility? (please specify the number)	Community health center : [ ][ ][ ]<br>Hospital-based laboratory : [ ][ ][ ]<br>Private clinic : [ ][ ][ ]		

GeneXpert Machine/Rapid Test Molecular (RTM)		
1.	When was the GeneXpert machine installed?	Date/Month/Year
2.	How many modules are there in the NAAT machine(s) at the laboratory?	☐ 4<br>☐ 8<br>☐ 16<br>Other:
3.	How many hours per week is the RTM machine operated?	_____hour(s)/week
4.	Is there a machine operation guideline available at the laboratory?	
5.	What is the number of laboratory personnel assigned to operate the machine and its computer?	☐ 1<br>☐ 2<br>☐ 3<br>Other:
**Supply chain and inventory**		

1.	Has there been any service disruption due to cartridge stock-out in the past 3 months?	☐ Yes<br>☐ No
	If "Yes", for how many days was RTM testing disrupted?	____day(s)
2.	Has any expired cartridge been found in the past 3 months?	☐ Yes<br>☐ No<br>*If "Yes", what is the estimated number of expired kits? (please specify the number)*<br>[ ][ ][ ] [ ]
3.	Where do you request cartridge stock?	☐ Local health office<br>☐ Government pharmacy and medical devices unit<br>☐ Other: ____________________________
4.	What tool/system is used to monitor cartridge stock?	☐ Inventory stock card<br>☐ Manual logbook<br>☐ Online logbook<br>☐ Laboratory information system<br>☐ Other:____________________________
5.	Is there a standard procedure or guideline for handling damaged and expired products?	☐ Yes<br>☐ No
**Maintenance**		
1.	Is the machine currently operating well?	☐ Yes<br>☐ No<br>☐ Several modules are not working properly; please specify the number ____
2.	What has been the average frequency of module failures over the past 12 months? *(please specify the number)*	
3.	When was the machine last calibrated?	[ ][ ]-[ ][ ]-[ ][ ]<br>Date Month Year
4.	When was the machine last maintained or repaired?	[ ][ ]-[ ][ ]-[ ][ ]<br>Date Month Year
5.	Are cleaning and maintenance records for the machine available?	☐ Yes<br>☐ No
	**Machine Utilization**	
1.	What is the number of samples tested in the last 3 months?	[ ][ ][ ] sample(s)
2.	What is the number of rejected samples in the past 3 months?	[ ][ ][ ] sample(s)<br>Please specify the reason(s):<br>1.______________________<br>2. _____________________<br>3.
3.	What is the number of samples detected as MTB RIF Sensitive in the last 3 months?	[ ][ ][ ] sample(s)
4.	What is the number of samples detected as MTB RIF Resistant in the last 3 months?	[ ][ ][ ] sample(s)
5.	What is the number of samples detected as MTB RR indeterminate in the last 3 months?	[ ][ ][ ] sample(s)
6.	What is the number of error results in the past 3 months?	[ ][ ][ ] sample(s)

7.	What is the number of invalid results in the past 3 months?	[ ][ ][ ] sampel(s)
8.	What is the number of tests with no result in the past 3 months?	
9.	Among the detected TB-Rif resistant samples, how many were from patients with no prior TB history, were subsequently retested, and what were the results?	____TB-Rifampicin resistant with no prior TB history<br>____retest<br>____TB-Rif resistant<br>____TB-SS
10.	Among the samples with TB-Rif resistant indeterminate results, how many were retested and what were the outcomes?	____retest<br>____TB-Rifampicin resistant<br>TB-SS
11.	What is the average turnaround time for testing samples using the TCM method (calculated from sample receipt to result release)?	[ ][ ] day(s)

**3.**	**Organization and management of the laboratory**		
1.	Service hours	[ ] Monday to Friday<br>[ ] Monday to Saturday<br>[ ] Everyday<br>[ ] Other:	
2.	In an outbreak situation, is there a mechanism to work in shifts?	[ ] Yes<br>[ ] No	
3.	Is the laboratory equipped with internet access?	[ ] Yes<br>[ ] No	
4.	Is there an adequate budget assigned for consumable and reagent purchase?	[ ] Yes<br>[ ] No	*If "Yes", please indicate the source of funds:*
5.	Is there an adequate budget assigned for equipment purchase/maintenance?	[ ] Yes<br>[ ] No	*If "Yes", please indicate the source of funds:*
6.	Has the laboratory been licensed (i.e. authorized to operate) by the authorities or hold any form of accreditation?	KALK (national) [ ] Yes [ ] No<br>ISO [ ] Yes [ ] No<br>**If yes, please give the details:**<br>1. …………………………………..<br>2. …………………………………..	
**4.**	**Human Resources**		
**A.**	**General Informations**		
1.	Number of:		
	a. Senior manager	[ ][ ]	
	b. Laboratory technician	[ ][ ]	
	c. Laboratory assistant	[ ][ ]	
	d. Administration staff	[ ][ ]	

	e. IT Staff	[ ][ ]		
2.	Is the staff number adequate to undertake biomolecular testing?	☐ Yes<br>☐ No		
3.	Is there any plan to increase the number of staff?	☐ Yes<br>☐ No		
**B.**	**Qualifications**	**Yes**	**No**	**Notes**
1.	Is there a periodic assessment of staff competency?			
2.	Has a quality manager been designated?			
3.	Has a biosafety officer been designated?			
4.	Is there quality management training in place for the staff?			
5.	Is there biosafety training in place for the staff?			If "Yes", check relevant box/es:<br>☐ risk assessment<br>☐ biosafety when handling sample<br>☐ use of disinfectant<br>☐ management of biological material<br>☐ management of hazardous and nonhazardous waste
6.	Is there biomolecular training in place for laboratory technicians?			If "Yes", check relevant box/es:<br>☐ nucleic acid extraction<br>☐ conventional PCR<br>☐ real-time PCR<br>☐ primer design<br>☐ PCR assay optimizing and validation
7.	Is there a professional development programme in place for the staff?			

**5.**	**Facilities**			
A.	**Infrastructure**	**Yes**	**No**	**Notes**
1.	Do you have an emergency electric generator or other backup power source?			If yes, please describe below:
2.	Does the building have adequate security to protect the equipment and computer from theft?			If yes, please describe below:
3.	Are emergency plans available (e.g. in case of explosion, fire, flood, worker exposure, accident or illness, major spillage)?			
4.	Is the room temperature well maintained? (the room where the diagnostic machine operates must be equipped with air conditioning and/or heating to ensure the temperature remains within the 15-30°C).			
5.	Are the storage room for reagents/kits with adequate temperature control available in place?			
6.	Are procedures for a safe and secure transport of culture, specimens,			

	samples and other contaminated materials established?			
7.	Is an information system related to biomolecular testing available in a place? (Check relevant box/es)	**Yes**	**No**	**Notes**
	a. Recording system			
	b. Reporting system			
	c. Notification system			
	d. Referral system			
**B.**	**Room for Biomolecular testing**	**Yes**	**No**	**Notes**
1.	Are there appropriate room(s) for molecular testing?			Please specify the number:
2.	Is the area of the room adequate to perform all testing activities?			Please specify the area:
3.	Is there an effective separation room for nucleic acid amplification testing (If relevent)?			
	a. Reagents preparation			
	b. Extraction			
	c. Amplification			
**C.**	**Work conditions**			**Notes**
1.	Does the laboratory face water shortages?	☐ Regularly<br>☐ Sometimes<br>☐ Seldom<br>☐ Never		
2.	Are work areas clean and well maintained?	☐ Yes<br>☐ No		
3.	Is sample collection carried out in room(s) separated from the laboratory examination room(s)?	☐ Yes<br>☐ No		
4.	Is there an effective separation between adjacent laboratory sections in which there are incompatible activities (e.g. nucleic acid extraction vs amplification)?	☐ Yes<br>☐ No		
5.	Are there designated rooms for specialized testing (TB, brucellosis, etc.)?	☐ Yes<br>☐ No		
6.	Are there appropriate storage areas?	☐ Yes<br>☐ No<br>If yes, specify the amount and describe the condition below:		

6.	Equipment			
**A.**	**Equipment inventory**	**Yes**	**No**	**Notes**
1.	Is there an equipment inventory for PCR machines and tools?			If yes or partial, does this form include:<br>☐ Name of the equipment<br>☐ Name and contact details of manufacturer<br>☐ Condition (i.e. new, used)<br>☐ Maintenance activities
**B.**	**Equipment maintenance, calibration and monitoring**			
1.	Does the laboratory have a dedicated person in charge of the equipment (maintenance management, etc.)?	☐ Yes<br>☐ No		
2.	Is the equipment maintained in a safe working condition (including electrical safety)?	☐ Yes<br>☐ No		
3.	Is there daily monitoring and recording of temperatures for temperature-dependent equipment?	☐ Yes<br>☐ No		
4.	Is there a defined protocol and time period for pipette calibration?	☐ Yes<br>☐ No		
5.	Is the staff duly trained and authorized before first using equipment?	☐ Yes<br>☐ No		

7.	**Consumables and reagents**		
**A.**	**Procurement**		
1.	Is there a responsible staff for consumable and reagent management (inventory, order, etc.)?	Yes<br>No	
2.	Does the laboratory experience problems with reagent delivery?		
	a. Delays	Never<br>Seldom<br>Sometimes<br>Regularly	
	b. Temperature not adequate	Never<br>Seldom<br>Sometimes<br>Regularly	
	c. Reference error	Never<br>Seldom<br>Sometimes<br>Regularly	
**B.**	**Inventory and Storage**		
1.	Is there an inventory system for consumables and reagents?	Yes<br>No	
2.	Are consumables and reagents inspected upon receipt?	Yes<br>No	
3.	Are consumables and reagents appropriately stored (temperature, humidity, etc.)?	Yes<br>No	
**C.**	**Use**		
1.	Is any expired reagent being used	Yes<br>No	*If "Yes", please decribe*:<br>Regularly<br>Sometimes<br>Seldom
2.	Is there a system for accurately forecasting needs for consumables and reagents?	Yes<br>No	

8.	Specimen Collection, Handling, and Transport	
A.	Specimen Collection	
1.	Are collection procedures documented and available to relevant personnel?	Yes<br>No
2.	Do these include minimum patient samples?	Yes<br>No
3.	Are there standards for collected specimens to be accepted for testing?	Yes<br>No
C.	Specimen Recording	
1.	Is a specimen recording system available in place?	Yes<br>No
2.	Are specimen portions traceable to the original primary sample (identification number, etc.)?	Yes<br>No
D.	Specimen Handling	
1.	Are primary specimens adequately stored at the recommended temperature?	Yes<br>No
2.	Are standardized procedures available in place for specimen storage after analysis?	Yes<br>No
D.	Specimen Referral/Transport	
1.	Is transportation system available in place for refering specimen?	Yes<br>No
2.	Is/are the person/s in charge of shipments trained for the transport of infectious substances?	Yes<br>No<br>[ ] He/She trained for local or national regulations<br>[ ] He/She trained in international regulations

9.	Biorisk Management			
A.	Biosafety Level	Yes	No	Notes
	1. BSL 1			
	2. BSL 2			
	3. Other:			
B.	Biosafety Management	Yes	No	Notes
1.	Are written biosafety procedures available for disinfection and decontamination?			
2.	Are written biosafety procedures available for disposal of infectious and non-infectious waste?			
3.	Is wastewater management facility available in place?			

10.	Data and Information Management			
A.	**Test Results and Reports**	**Ye s**	**No**	**Notes**
1.	Are all original observations/results of the laboratory recorded in a worksheet or electronic database?			
2.	Are the results reviewed and authorized before the results are released?			
3.	When samples need to be referred further to another laboratory, is there procedure to define how a report is then issued?			
B.	**Data Analysis and Statistics**	**Ye s**	**No**	**Notes**
1.	Can the laboratory provide basic statistical data (e.g. number of tests ordered, aggregated qualitative/quantitative data, etc.)?			
C.	**Data Security and Confidentiality**	**Ye s**	**No**	**Notes**
1.	Are access and modification of patient data protected?			
2.	Is efficient back-up in place to prevent loss of patient result data in case of theft or other incident at the laboratory?			
D.	**IT and Laboratory Information System (LIS)**	**Ye s**	**No**	**Notes**
1.	Is a Laboratory Information System available in place?			

11.	Biomolecular staff statements regarding PCR diagnostic testing	SD	D	N	A	SA
1.	Open PCR diagnostic method is difficult to do					
2.	Open PCR diagnostic method is troublesome					
3.	Open PCR diagnostic method needs trained personnel to do					
4.	Ready to use open PCR diagnostic method if it is available in the laboratory					

SD: Strongly Disagree, D: Disagree, N: Neutral; A: Agree, SA: Strongly Agree

Preliminary Questionnaire

Laboratory Preliminary Questionnaire		
1.	Name	
2.	Telephone	
3.	Name of the laboratory	
4.	District/City	
5.	Address	
6.	Level of laboratory	☐ Tier 1 (Peripheral)<br>☐ Tier 2 (District)<br>☐ Tier 3 (Intermediate)<br>☐ Tier 4 (Regional)<br>☐ Tier 5 (Central)
7.	Status of laboratory	☐ BLUD<br>☐ Non BLUD
8.	Is PCR COVID-19 testing available in place?	[ ] Yes (go to question 10)<br>[ ] No (go to question 9)
9.	Does the laboratory still possess NAAT/PCR machines used for COVID-19 test in the pandemic?	[ ] Yes<br>[ ] No
10.	Has the available NAAT/PCR machine ever been used for COVID-19 testing?	[ ] Yes<br>[ ] No
11.	Status of PCR machine procurement	[ ] Local government budget<br>[ ] National government budget<br>[ ] Grant<br>If "Grant", please describe below:
12.	What brand(s) is/are the available PCR machines in your laboratory?	
13.	Type of PCR machine	[ ] Open system<br>[ ] Closed system
14.	Type of reagent used for PCR testing in your laboratory?	
15.	What is the maximum capacity for each test cycle? (in number)	_____samples
16.	What is the duration for each test?	[ ] [ ] [ ] [ ] ____/test
17.	How long is the PCR machine being operated daily?	_____hours/day
18.	Does your laboratory possess any of these following GeneXpert machines?	[ ] GeneXpert MTB/Rif Test<br>[ ] GeneXpert MTB/Rif Ultra<br>[ ] GeneXpert MTB/Rif XDR<br>[ ] None
19.	When was the GeneXpert machine(s) installed?	dd/mm/yyyy
20.	How many modules does your GeneXpert machine have?	[ ] 4<br>[ ] 8<br>[ ] 16

21.	How long is GeneXpert machine being operated weekly?	____hours/week
