# Supplementary material for: Assessment of post-pandemic NAAT-based diagnostic capacity among laboratories with COVID-19 testing resource investments in Indonesia
Source: PLoS One. 2026 Apr 2;21(4):e0343628. doi: 10.1371/journal.pone.0343628 (PMC13046156; doi:10.1371/journal.pone.0343628)
Supplement: S2 File — (PDF) [file pone.0343628.s002.pdf]

Supporting Information 2 – Scoring of Laboratory Assessment Tool Questionnaire

1. Organization and Management Module

DHL	Availability of Internet Access	Availability of Sufficient Budget for Reagents and Consumables	Availability of Sufficient Budget for Tools' Purchase and Maintenance	Nationally Accredited (KALK)	Internationally Accredited (ISO)	Percentage per DHL
**DHL 1**	1	1	0	1	0	60.0
**DHL 2**	1	1	1	1	0	80.0
**DHL 3**	1	0	0	1	0	40.0
**DHL 4**	1	1	1	1	0	80.0
**DHL 5**	1	1	1	1	0	80.0
**DHL 6**	1	0	1	1	1	80.0
**DHL 7**	1	1	1	1	1	100.0
**DHL 8**	1	1	0	0	0	40.0
**DHL 9**	1	1	1	1	1	100.0
**DHL 10**	1	1	0	0	0	40.0
**DHL 11**	1	1	1	1	0	80.0
**DHL 12**	1	1	0	1	0	60.0
**DHL 13**	1	1	1	1	0	80.0
**DHL 14**	1	1	1	1	0	80.0
**DHL 15**	1	1	1	1	0	80.0
**DHL 16**	1	0	0	1	1	60.0
**DHL 17**	1	0	0	1	1	60.0
**DHL 18**	1	1	1	1	1	100.0
**DHL 19**	1	1	1	1	0	80.0
**DHL 20**	1	1	1	1	0	80.0
**DHL 21**	1	1	1	0	0	60.0
**DHL 22**	1	0	0	1	0	40.0
**DHL 23**	1	0	0	1	0	40.0
**DHL 24**	1	0	0	1	0	40.0

**DHL 25**	1	0	0	1	1	60.0
**DHL 26**	1	0	0	1	0	40.0
**Percentage per question**	100	65.4	53.8	88.5	26.9	66.9

2. Human Resources Module

DHL	Provision of Molecular Testing Training	Provision of PCR training	Presence of Quality Manager	Provision of Quality Management Training	Availability of Routine Personnels' Assessments	Availability of staff with biomolecular testing capacity	Provision of Staff Professional Development Efforts	Percentage per DHL
**DHL 1**	1	1	1	1	1	1	1	100.0
**DHL 2**	1	1	1	1	1	1	1	100.0
**DHL 3**	1	1	1	0	1	1	1	85.7
**DHL 4**	1	0	1	1	1	1	1	85.7
**DHL 5**	0	0	1	1	1	0	0	42.9
**DHL 6**	1	1	1	1	1	1	1	100.0
**DHL 7**	1	1	1	1	1	1	1	100.0
**DHL 8**	1	1	1	1	1	1	1	100.0
**DHL 9**	1	1	1	1	1	1	1	100.0
**DHL 10**	0	0	1	1	0	0	1	42.9
**DHL 11**	1	1	1	1	1	1	1	100.0
**DHL 12**	1	1	1	1	1	1	1	100.0
**DHL 13**	0	0	1	1	1	0	1	57.1
**DHL 14**	1	0	1	1	0	1	1	71.4
**DHL 15**	1	1	1	1	1	1	1	100.0
**DHL 16**	1	1	1	1	1	1	1	100.0
**DHL 17**	1	1	1	1	1	1	1	100.0
**DHL 18**	1	1	1	1	0	1	1	85.7
**DHL 19**	1	1	1	1	1	1	1	100.0
**DHL 20**	1	1	1	0	1	1	1	85.7
**DHL 21**	0	0	1	1	1	0	0	42.9
**DHL 22**	1	1	1	1	1	1	1	100.0
**DHL 23**	1	1	1	1	1	1	0	85.7
**DHL 24**	0	0	1	1	1	0	1	57.1
**DHL 25**	1	1	1	1	1	1	1	100.0
**DHL 26**	0	0	1	0	0	0	1	28.6
**Percentage per question**	76.9	69.2	100	88.5	84.6	76.9	88.5	83.5

3. Facilities Module

DHL	Availability of BSL-2 facility	BSL-2 remains functional	Stable electricity	Availability of Electricity Back-up	Presence of Specific Security Personnel for Lab Building	Presence of Security Alarm	Presence of CCTV	Controlled Room Temperature	Availability of Reagents Storage Room	Availability of Molecular Testing Room	Availability of Standardized Separate Rooms for	Water Shortage	Well-maintained bench	Availability of Specialized Room for Sample Collection	Availability of Specialized Room for Tuberculosis Testing	Availability of regular refrigerator/chiller	Availability of -20oC Freezer	Availability of -80oC Freezer	Percentage per DHL
**DHL 1**	1	1	1	1	0	0	1	1	1	1	1	1	1	1	0	0	1	1	77.8
**DHL 2**	0.5	0	1	1	0	1	1	1	1	1	1	1	1	1	0	1	1	1	80.6
**DHL 3**	1	1	1	1	0	0	1	1	1	1	1	1	1	1	1	1	0	1	83.3
**DHL 4**	1	1	1	1	0	1	0	1	1	1	1	0.67	1	1	0	0	0	0	64.8
**DHL 5**	0	0	1	1	0	0	0	1	1	1	0	1	1	0	0	0	0	0	38.9
**DHL 6**	0.5	0	1	1	1	0	1	1	1	1	1	1	1	1	0	1	1	0	75.0
**DHL 7**	1	1	1	1	0	0	0	1	1	1	1	1	1	1	1	1	0	0	72.2
**DHL 8**	1	1	1	1	0	1	0	1	1	1	1	1	1	1	1	1	1	0	83.3
**DHL 9**	1	1	1	1	0	0	0	1	1	1	1	1	1	1	1	1	1	1	83.3
**DHL 10**	1	1	1	1	1	0	1	1	1	1	1	1	1	1	1	1	1	0	88.9
**DHL 11**	1	0	1	0	0	0	0	0	1	0	1	1	0	1	1	0	0	0	38.9
**DHL 12**	1	1	0	1	0	0	1	1	1	1	1	0.67	1	1	0	1	1	0	70.4
**DHL 13**	0	0	1	1	1	0	0	1	1	0	0	1	1	1	0	1	0	0	50.0
**DHL 14**	0	0	1	1	1	0	0	1	1	0	0	1	0	0	0	0	0	0	33.3

**DHL 15**	0.5	0	1	1	1	0	0	1	0	1	0	1	1	1	1	1	0	0	58.3
**DHL 16**	1	0	1	1	0	0	0	0	1	0	1	1	0	1	0	1	1	1	55.6
**DHL 17**	1	1	1	1	1	1	1	1	1	1	1	1	1	1	1	1	1	1	100.0
**DHL 18**	0.5	0	1	1	1	0	1	1	1	1	0	1	1	1	1	1	1	1	80.6
**DHL 19**	1	0	1	1	0	0	0	0	1	1	1	1	1	1	0	1	0	0	55.6
**DHL 20**	1	1	1	1	1	1	1	1	1	1	1	1	1	1	0	1	1	0	88.9
**DHL 21**	0.5	0	0	0	0	0	0	0	0	0	0	1	0	0	0	1	1	0	19.4
**DHL 22**	1	0	0	1	0	0	1	1	1	1	1	1	1	1	1	1	0	0	66.7
**DHL 23**	1	1	1	1	1	1	1	1	1	1	1	1	1	1	0	1	1	0	88.9
**DHL 24**	1	0	1	1	1	0	0	1	1	1	1	1	1	0	0	1	1	1	72.2
**DHL 25**	1	1	1	1	1	0	0	1	1	1	0	1	1	1	1	1	0	0	72.2
**DHL 26**	0.5	0	1	1	0	0	0	0	1	0	0	0.33	0	0	0	1	0	0	26.8
**Percentage per question**	76.9	46.2	88.5	92.3	42.3	23.1	42.3	80.8	92.3	76.9	69.2	94.9	80.8	80.8	42.3	80.8	53.8	30.8	66.4

4. Equipment Module

DHL	Presence of Machines' Names Inventory List	Presence of Machines' Manufacturer Details Inventory List	Presence of Machines' Conditions Inventory List	Presence of General Maintenance Activities	Presence of Dedicated Staff Responsible for PCR Machine	All Equipments are Mainted in Proper Electrical and Temperature Condition	Presence of Temperature Monitoring and Recording Routine	Availability of Calibration Protocol and Specific Schedule	Provision of Training Prior to Machines Use	Percentage per DHL
DHL 1	1	1	1	1	1	1	1	1	1	100.0
DHL 2	1	1	1	1	1	1	1	1	1	100.0
DHL 3	0	0	0	0	1	0	1	0	1	33.3
DHL 4	0	0	0	0	1	1	1	1	1	55.6
DHL 5	1	1	0	0	1	1	0	1	1	66.7
DHL 6	1	1	1	1	1	1	1	1	1	100.0
DHL 7	1	1	1	1	1	0	1	0	1	77.8
DHL 8	1	0	1	0	1	0	1	0	1	55.6
DHL 9	1	1	0	0	1	1	1	1	1	77.8
DHL 10	1	1	1	1	1	1	1	0	0	77.8
DHL 11	0	0	0	0	1	1	1	1	1	55.6
DHL 12	1	1	1	1	1	1	1	1	1	100.0
DHL 13	1	1	1	1	1	1	1	1	0	88.9
DHL 14	1	1	1	1	1	0	0	0	1	66.7
DHL 15	1	1	1	1	1	1	1	1	1	100.0
DHL 16	1	1	0	0	0	0	0	0	1	33.3
DHL 17	1	1	1	1	1	1	1	1	1	100.0
DHL 18	1	1	1	1	1	1	1	1	1	100.0
DHL 19	1	1	1	0	1	1	0	0	1	66.7
DHL 20	1	1	1	1	1	1	1	1	1	100.0
DHL 21	1	1	1	1	1	0	1	1	0	77.8
DHL 22	1	1	1	1	1	0	1	1	1	88.9
DHL 23	1	1	1	0	1	1	1	1	1	88.9

**DHL 24**	1	1	1	1	1	1	1	1	0	88.9
**DHL 25**	1	1	1	1	1	1	1	1	1	100.0
**DHL 26**	0	0	0	0	0	0	0	0	0	0.0
**Percentage per question**	84.6	80.8	73.1	61.5	92.3	69.2	80.8	69.2	80.8	76.9

5. Consumables Module

DHL	Availability of Person In Charge for Consumables and Reagents	Frequency of Delay in Consumables Shipment	Frequency of Recorded Poor Temperature	Frequency of Reference Error	Availability of Consumables and Reagents Inventory List	Checking of Consumables and Reagents after Being Delivered	Storage of Consumables and Reagents with Monitored Temperature	Avoidance of Use of Expired Reagent	Availability of Working System to Predict Consumables and Reagents Need	Percentage per DHL
DHL 1	1	1	1	1	1	1	1	1	1	100.0
DHL 2	1	0.33	1	1	1	1	1	1	1	92.6
DHL 3	1	1	1	1	1	1	1	1	1	100.0
DHL 4	1	0.67	1	0.33	1	1	0	1	0	66.7
DHL 5	1	1	1	0.33	1	1	1	1	1	92.6
DHL 6	1	1	0.67	1	1	1	1	1	1	96.3
DHL 7	1	1	1	1	1	1	1	1	1	100.0
DHL 8	1	0	1	0.67	1	1	1	1	0	74.1
DHL 9	1	1	0.67	1	1	1	1	1	0	85.2
DHL 10	1	1	0.67	1	1	1	1	1	1	96.3
DHL 11	1	1	1	1	1	1	1	1	1	100.0
DHL 12	1	0.67	0.67	1	1	1	1	1	1	92.7
DHL 13	1	0.67	1	0.67	1	1	1	1	1	92.7
DHL 14	1	1	1	1	1	1	1	1	1	100.0
DHL 15	1	1	1	0.67	1	1	0	1	1	85.2
DHL 16	1	0.33	1	0.67	1	1	1	1	1	88.9
DHL 17	1	0.67	0.67	0.67	1	1	1	1	1	89.0
DHL 18	1	1	1	0.67	1	1	1	1	1	96.3
DHL 19	1	1	1	1	1	1	0	1	1	88.9
DHL 20	1	0.67	0.67	1	1	1	1	1	1	92.7
DHL 21	1	1	1	1	1	1	1	1	1	100.0
DHL 22	1	1	1	1	1	1	1	1	1	100.0
DHL 23	1	1	1	1	1	1	1	1	1	100.0

**DHL 24**	1	1	1	0.67	1	1	1	1	1	96.3
**DHL 25**	1	0.67	1	0.67	1	1	1	1	1	92.7
**DHL 26**	1	0.67	1	0.67	1	1	1	1	1	92.7
**Percentage per question**	100	82.1	92.4	83.4	100	100	88.5	100	88.5	92.8

6. Specimen Collection, Handling, and Transport Module

DHL	Availability of operational procedure for specimen collection and access by dedicated staff	Availability of Specimen Collection Standard to be Accepted for Testing	Availability of specimen recording into lab's system	Storage of specimen at recommended temperature	Availability of standardized procedure for specimen handling after analysis	Percentage per DHL
DHL 1	1	1	1	0	1	80.0
DHL 2	1	1	1	1	0	80.0
DHL 3	1	1	1	1	1	100.0
DHL 4	0	0	0	0	0	0.0
DHL 5	0	0	0	0	0	0.0
DHL 6	1	1	1	1	1	100.0
DHL 7	1	1	1	1	1	100.0
DHL 8	0	1	1	1	1	80.0
DHL 9	1	1	1	1	1	100.0
DHL 10	0	0	0	0	0	0.0
DHL 11	1	1	1	0	1	80.0
DHL 12	1	1	1	1	1	100.0
DHL 13	0	0	0	0	0	0.0
DHL 14	1	1	1	0	0	60.0
DHL 15	1	1	1	1	1	100.0
DHL 16	0	1	1	1	1	80.0
DHL 17	1	1	1	1	1	100.0
DHL 18	1	1	1	1	1	100.0
DHL 19	1	1	1	1	1	100.0
DHL 20	1	1	1	1	1	100.0
DHL 21	0	0	0	0	0	0.0
DHL 22	1	1	1	1	1	100.0
DHL 23	1	1	1	1	1	100.0
DHL 24	1	1	1	1	1	100.0

**DHL 25**	1	1	1	1	1	100.0
**DHL 26**	0	0	0	0	0	0.0
**Percentage per question**	69.2	76.9	76.9	65.4	69.2	71.5

7. **Biorisk Management Module**

DHL	Availability of Disaster Contingency Plan	Presence of Biosafety Officer	Provision of Biosafety Training	Availability of Biosafety Operational Procedure for Testing	Availability of Standardized Procedure for Disinfection and Decontamination	Availability of Standardized Procedure for Managing Infectious and Non-Infectious Wastes	Availability of Wastewater Management Installation	Percentage per DHL
**DHL 1**	1	1	1	1	1	1	1	100.0
**DHL 2**	1	0	1	1	1	1	1	85.7
**DHL 3**	1	0	0	1	1	1	1	71.4
**DHL 4**	1	1	0	1	1	1	1	85.7
**DHL 5**	1	1	0	0	1	1	1	71.4
**DHL 6**	1	1	1	1	1	1	1	100.0
**DHL 7**	1	0	0	1	1	1	1	71.4
**DHL 8**	1	1	1	1	1	1	1	100.0
**DHL 9**	0	1	1	1	1	1	1	85.7
**DHL 10**	1	1	0	0	1	1	1	71.4
**DHL 11**	0	1	0	1	1	1	1	71.4
**DHL 12**	1	1	0	1	0	1	0	57.1
**DHL 13**	1	1	0	0	1	1	1	71.4
**DHL 14**	1	1	1	1	0	1	1	85.7
**DHL 15**	1	1	1	1	1	1	1	100.0
**DHL 16**	1	1	1	1	1	1	1	100.0
**DHL 17**	1	1	1	1	1	1	1	100.0
**DHL 18**	1	1	1	1	1	1	1	100.0
**DHL 19**	1	1	1	0	1	1	1	85.7
**DHL 20**	1	1	1	1	1	1	1	100.0
**DHL 21**	1	1	1	0	1	1	1	85.7
**DHL 22**	1	1	0	1	1	1	1	85.7
**DHL 23**	1	1	1	1	1	1	1	100.0

**DHL 24**	1	1	1	0	1	1	0	71.4
**DHL 25**	1	1	1	1	1	1	1	100.0
**DHL 26**	1	1	1	1	1	1	1	100.0
**Percentage per question**	92.3	88.5	65.4	76.9	92.3	100	92.3	86.8

8. Data and Information Management Module

DHL	Availability of Lab Results Database	Review of Lab Test Results Before Disseminated	Availability of Reporting Procedures for Referred Samples	Standard Statistical Evaluation of Testing Provision and Results	Access protection for patients' data	Availability of data back-up mechanism	Availability of Laboratory Information System	Percentage per DHL
**DHL 1**	1	1	1	1	1	1	1	100.0
**DHL 2**	1	1	1	1	1	1	0	85.7
**DHL 3**	1	1	1	1	1	1	0	85.7
**DHL 4**	1	1	1	1	1	0	1	85.7
**DHL 5**	1	1	1	1	1	1	0	85.7
**DHL 6**	1	1	0	0	1	0	0	42.9
**DHL 7**	1	1	1	1	1	1	1	100.0
**DHL 8**	1	1	1	0	1	0	0	57.1
**DHL 9**	1	1	1	1	0	1	0	71.4
**DHL 10**	1	1	1	1	1	0	0	71.4
**DHL 11**	1	0	1	0	1	1	0	57.1
**DHL 12**	1	1	0	0	1	1	0	57.1
**DHL 13**	1	1	1	1	1	0	0	71.4
**DHL 14**	1	1	1	0	1	1	0	71.4
**DHL 15**	1	1	1	1	1	1	0	85.7
**DHL 16**	1	1	0	1	1	1	1	85.7
**DHL 17**	1	1	1	1	1	1	0	85.7
**DHL 18**	1	1	1	1	1	1	0	85.7
**DHL 19**	1	1	1	1	1	0	1	85.7
**DHL 20**	1	1	1	1	1	1	0	85.7
**DHL 21**	1	1	0	1	0	1	0	57.1
**DHL 22**	1	1	1	1	1	1	0	85.7
**DHL 23**	1	1	1	1	1	1	0	85.7
**DHL 24**	1	1	0	0	1	0	0	42.9
**DHL 25**	1	1	1	1	1	1	1	100.0
**DHL 26**	1	1	0	0	1	0	1	57.1
**Percentage per question**	100	96.2	76.9	73.1	92.3	69.2	26.9	76.4

9. Lab Testing Performance Module (PCR)

DHL	Availability of Qualified Staff to Operate the Machine	Availability of the Machine	Availability of Procedure for Machine Use	Availability of Non-Expired Reagents	Machine has been calibrated in the past year	Availability of general maintenance of the machine	Percentage per DHL
**DHL 1**	1	1	1	1	0	1	83.3
**DHL 2**	1	1	1	0	0	1	66.7
**DHL 3**	1	1	1	0	0	0	50.0
**DHL 4**	1	1	1	0	0	0	50.0
**DHL 5**	1	1	0	0	0	0	33.3
**DHL 6**	1	1	1	0	0	1	66.7
**DHL 7**	1	1	1	1	0	1	83.3
**DHL 8**	0	1	1	0	0	0	33.3
**DHL 9**	1	1	1	1	1	0	83.3
**DHL 10**	0	1	0	0	0	1	33.3
**DHL 11**	1	1	1	0	0	0	50.0
**DHL 12**	1	1	1	0	0	1	66.7
**DHL 13**	1	1	0	0	1	1	66.7
**DHL 14**	1	1	1	0	0	1	66.7
**DHL 15**	1	1	1	0	0	1	66.7
**DHL 16**	1	1	1	0	0	0	50.0
**DHL 17**	1	1	1	0	0	1	66.7
**DHL 18**	1	1	1	0	0	1	66.7
**DHL 19**	1	1	0	1	0	0	50.0
**DHL 20**	1	1	1	0	1	1	83.3
**DHL 21**	0	1	0	0	0	1	33.3
**DHL 22**	1	1	1	0	0	1	66.7
**DHL 23**	1	1	1	0	0	0	50.0
**DHL 24**	1	1	0	0	0	1	50.0
**DHL 25**	1	1	1	1	0	1	83.3
**DHL 26**	0	0	0	0	0	0	0.00
**Percentage per question**	84.6	96.2	73.1	19.2	11.5	61.5	57.7

Total Calculation of All Modules

DHL	Organization and Management	Human Resources	Facilities	Equipment	Consumables	Specimen handling	Biorisk management	Data and information management	Lab Testing Performance (PCR)	Final Laboratory Capacity
**DHL 1**	60.0	100.0	77.8	100.0	100.0	80.0	100.0	100.0	83.3	89.0
**DHL 2**	80.0	100.0	80.6	100.0	92.6	80.0	85.7	85.7	66.7	85.7
**DHL 3**	40.0	85.7	83.3	33.3	100.0	100.0	71.4	85.7	50.0	72.2
**DHL 4**	80.0	85.7	64.8	55.6	66.7	0.0	85.7	85.7	50.0	63.8
**DHL 5**	80.0	42.9	38.9	66.7	92.6	0.0	71.4	85.7	33.3	56.8
**DHL 6**	80.0	100.0	75.0	100.0	96.3	100.0	100.0	42.9	66.7	84.5
**DHL 7**	100.0	100.0	72.2	77.8	100.0	100.0	71.4	100.0	83.3	89.4
**DHL 8**	40.0	100.0	83.3	55.6	74.1	80.0	100.0	57.1	33.3	69.3
**DHL 9**	100.0	100.0	83.3	77.8	85.2	100.0	85.7	71.4	83.3	87.4
**DHL 10**	40.0	42.9	88.9	77.8	96.3	0.0	71.4	71.4	33.3	58.0
**DHL 11**	80.0	100.0	38.9	55.6	100.0	80.0	71.4	57.1	50.0	70.3
**DHL 12**	60.0	100.0	70.4	100.0	92.7	100.0	57.1	57.1	66.7	78.2
**DHL 13**	80.0	57.1	50.0	88.9	92.7	0.0	71.4	71.4	66.7	64.2
**DHL 14**	80.0	71.4	33.3	66.7	100.0	60.0	85.7	71.4	66.7	70.6
**DHL 15**	80.0	100.0	58.3	100.0	85.2	100.0	100.0	85.7	66.7	86.2
**DHL 16**	60.0	100.0	55.6	33.3	88.9	80.0	100.0	85.7	50.0	72.6
**DHL 17**	60.0	100.0	100.0	100.0	89.0	100.0	100.0	85.7	66.7	89.0
**DHL 18**	100.0	85.7	80.6	100.0	96.3	100.0	100.0	85.7	66.7	90.6
**DHL 19**	80.0	100.0	55.6	66.7	88.9	100.0	85.7	85.7	50.0	79.2
**DHL 20**	80.0	85.7	88.9	100.0	92.7	100.0	100.0	85.7	83.3	90.7
**DHL 21**	60.0	42.9	19.4	77.8	100.0	0.0	85.7	57.1	33.3	52.9
**DHL 22**	40.0	100.0	66.7	88.9	100.0	100.0	85.7	85.7	66.7	81.5
**DHL 23**	40.0	85.7	88.9	88.9	100.0	100.0	100.0	85.7	50.0	82.1
**DHL 24**	40.0	57.1	72.2	88.9	96.3	100.0	71.4	42.9	50.0	68.8
**DHL 25**	60.0	100.0	72.2	100.0	92.7	100.0	100.0	100.0	83.3	89.8
**DHL 26**	40.0	28.6	26.8	0.0	92.7	0.0	100.0	57.1	0.00	38.4
**Average**										75.4
