# Supplementary material for: Assessment of post-pandemic NAAT-based diagnostic capacity among laboratories with COVID-19 testing resource investments in Indonesia
Source: PLoS One. 2026 Apr 2;21(4):e0343628. doi: 10.1371/journal.pone.0343628 (PMC13046156; doi:10.1371/journal.pone.0343628)
Supplement: S3 File — (PDF) [file pone.0343628.s003.pdf]

# Supporting File 3 : Descriptive statistics of all aspects from the assessment module (N=26)

| | Module | Aspect | Yes (n, %) |
|---|---|---|---|
| A. | Laboratory Management | Availability of internet access | 26 (100.0) |
| | | Sufficient budget assigned for consumable and reagent purchase | 17 (65.4) |
| | | Sufficient budget assigned for equipment purchase and maintenance | 14 (53.8) |
| | | Nationally accredited (KALK) | 23 (88.5) |
| | | Internationally accredited (ISO) | 7 (26.9) |
| B. | Human Resources | Staff qualification | **Median (IQR)** |
| | | - Laboratory technician | 7 (4 – 9) |
| | | - Trained staff to do PCR | 2 (1 – 4) |
| | | Training | **Yes (n, %)** |
| | | - Molecular testing | 20 (76.9) |
| | | - Real-time PCR | 18 (69.2) |
| | | - Quality management | 23 (88.5) |
| | | Provision of quality manager | 26 (100.0) |
| | | Availability of routine personnels' assessments | 22 (84.6) |
| | | Availability of staff with biomolecular testing capacity | 20 (76.9) |
| | | Provision of staff professional development efforts | 23 (88.5) |
| C. | Laboratory Facilities | Availability of BSL-2 laboratory | |
| | | - Yes | 17 (65.4) |
| | | - Under construction | 6 (23.1) |
| | | Functional BSL-2 laboratory | 12 (46.2) |
| | | Stable electricity | 23 (88.5) |
| | | Availability of electricity back-up | 24 (92.3) |
| | | Presence of specific security personnel for laboratory building | 11 (42.3) |
| | | Presence of security alarm | 6 (23.1) |
| | | Presence of CCTV | 11 (42.3) |
| | | Controlled room temperature | 21 (80.8) |
| | | Availability of reagents storage room | 24 (92.3) |
| | | Availability of molecular testing room | 20 (76.9) |
| | | Availability of standardized separate rooms for molecular testing room/tri-room separation (preparation, extraction, and amplification) | 18 (69.2) |
| | | Water shortage | |
| | | - Never | 23 (88.5) |
| | | - Seldom | 2 (7.7) |

| Module | | Aspect | Yes (n, %) |
|---|---|---|---|
| | | - Sometimes | 1 (3.8) |
| | | Well-maintained bench | 21 (80.8) |
| | | Availability of specialized room for sample collection | 21 (80.8) |
| | | Availability of specialized room for tuberculosis testing | 11 (42.3) |
| | | Availability of refrigerator/chiller | 21 (80.8) |
| | | Availability of freezer -20ºC | 14 (53.8) |
| | | Availability of freezer -80ºC | 8 (30.8) |
| **D.** | **Equipment Management** | Presence of machines' names inventory list | 22 (84.6) |
| | | Presence of machines' manufacturer details inventory list | 21 (80.8) |
| | | Presence of machines' conditions inventory list | 19 (73.1) |
| | | Presence of general maintenance activities | 16 (61.5) |
| | | Presence of dedicated staff responsible for PCR machine | 24 (92.3) |
| | | All equipment are maintained in proper electrical and temperature condition | 18 (69.2) |
| | | Presence of temperature monitoring and recording routine | 21 (80.8) |
| | | Availability of equipment calibration's protocol and scheduling | 18 (69.2) |
| | | Provision of training prior to machines use | 21 (80.8) |
| **E.** | **Specimen collection, handling, and transport** | Availability of operational procedure for specimen collection and access by dedicated staff | 18 (69.2) |
| | | Availability of specimen collection standard to be accepted for testing | 20 (76.9) |
| | | Availability of specimen recording into lab's system | 20 (76.9) |
| | | Storage of specimen at recommended temperature | 17 (65.4) |
| | | Availability of standardized procedure for specimen storage post-analysis | 18 (69.2) |
| **F.** | **Consumables and Reagents Management** | Availability of person in charge for consumables and reagents | 26 (100.0) |
| | | Frequency of delay in consumables shipment | |
| | | - Never | 16 (61.5) |
| | | - Seldom | 7 (26.9) |
| | | - Sometimes | 2 (7.7) |
| | | - Regularly | 1 (3.8) |
| | | Frequency of recorded poor temperature | |
| | | - Never | 20 (76.9) |
| | | - Seldom | 6 (23.1) |
| | | Frequency of reference error | |
| | | - Never | 15 (57.7) |
| | | - Seldom | 9 (34.6) |
| | | - Sometimes | 2 (7.7) |

| | Module | Aspect | Yes (n, %) |
|---|---|---|---|
| | | Availability of consumables and reagents inventory list | 26 (100.0) |
| | | Checking of consumables and reagents after being delivered | 26 (100.0) |
| | | Appropriate storage of consumables and reagents with temperature and humidity monitoring | 23 (88.5) |
| | | Avoidance of use of expired reagent | 26 (100.0) |
| | | Availability of a system for accurately forecasting needs for consumables and reagent | 23 (88.5) |
| **G.** | **Laboratory testing performance** | PCR: | |
| | | - Competent staff to perform the test | 22 (84.6) |
| | | - Availability of the machine | 25 (96.2) |
| | | - Availability of procedure for machine use | 19 (73.1) |
| | | - Availability of non-expired reagents | 5 (19.2) |
| | | - Machine calibration in the past year | 3 (11.5) |
| | | - Availability of general maintenance of the machine | 16 (61.5) |
| **H.** | **Biorisk Management** | Availability of disaster contingency plan | 24 (92.3) |
| | | Presence of biosafety officer | 23 (88.5) |
| | | Provision of biosafety training | 17 (65.4) |
| | | Availability of biosafety operational procedure for testing | 20 (76.9) |
| | | Availability of standardized procedure for disinfection and decontamination | 24 (92.3) |
| | | Availability of management procedure for infectious and non-infectious waste | 26 (100.0) |
| | | Availability of wastewater management installation | 24 (92.3) |
| **I.** | **Data and Information System** | Availability of lab results database | 26 (100.0) |
| | | Review of lab test results before disseminated | 25 (96.2) |
| | | Availability of reporting procedures for referred samples | 20 (76.9) |
| | | Standard statistical evaluation of testing provision and results | 19 (73.1) |
| | | Access protection for patient data | 24 (92.3) |
| | | Patient result data backup | 18 (69.2) |
| | | Availability of laboratory information system (LIS) | 7 (26.9) |

**PCR-trained Staff Perceptions Regarding Open PCR Diagnostic Method (N=22)**

| No | Statement | Frequency of agreement | N (%) |
|---|---|---|---|
| 1 | Open PCR diagnostic method is difficult to do | Disagree | 12 (54.6) |
| | | Neutral | 5 (22.7) |
| | | Strongly disagree | 3 (13.6) |
| | | Agree | 2 (9.1) |
| 2 | Open PCR diagnostic method is troublesome to do | Disagree | 10 (45.4) |
| | | Neutral | 6 (27.3) |
| | | Agree | 4 (18.2) |
| | | Strongly disagree | 2 (9.1) |
| 3 | Open PCR diagnostic method needs trained personnel to do | Strongly agree | 14 (63.6) |
| | | Agree | 6 (27.3) |
| | | Strongly disagree | 2 (9.1) |
| 4 | Readiness to use open PCR diagnostic method if it is available in Laboratory | Agree | 15 (68.2) |
| | | Strongly agree | 6 (27.3) |
| | | Strongly disagree | 1 (4.5) |
